# Personalized Explanations for Early Diagnosis of Alzheimer’s Disease Using Explainable Graph Neural Networks with Population Graphs

**DOI:** 10.3390/bioengineering10060701

**Published:** 2023-06-08

**Authors:** So Yeon Kim

**Affiliations:** 1 Department of Artificial Intelligence, Ajou University, Suwon 16499, Republic of Korea; jebi1771@ajou.ac.kr; 2 Department of Software and Computer Engineering, Ajou University, Suwon 16499, Republic of Korea

**Keywords:** graph neural networks, alzheimer’s disease, amyloid-beta positivity, population graph, explainable graph neural networks

## Abstract

Leveraging recent advances in graph neural networks, our study introduces an application of graph convolutional networks (GCNs) within a correlation-based population graph, aiming to enhance Alzheimer’s disease (AD) prognosis and illuminate the intricacies of AD progression. This methodological approach leverages the inherent structure and correlations in demographic and neuroimaging data to predict amyloid-beta (Aβ) positivity. To validate our approach, we conducted extensive performance comparisons with conventional machine learning models and a GCN model with randomly assigned edges. The results consistently highlighted the superior performance of the correlation-based GCN model across different sample groups in the Alzheimer’s Disease Neuroimaging Initiative (ADNI) dataset, suggesting the importance of accurately reflecting the correlation structure in population graphs for effective pattern recognition and accurate prediction. Furthermore, our exploration of the model’s decision-making process using GNNExplainer identified unique sets of biomarkers indicative of Aβ positivity in different groups, shedding light on the heterogeneity of AD progression. This study underscores the potential of our proposed approach for more nuanced AD prognoses, potentially informing more personalized and precise therapeutic strategies. Future research can extend these findings by integrating diverse data sources, employing longitudinal data, and refining the interpretability of the model, which potentially has broad applicability to other complex diseases.

## 1. Introduction

Alzheimer’s disease (AD) is a progressive neurodegenerative disorder characterized by a series of changes in the brain that occur years or even decades before the first symptoms of cognitive decline become evident [1,2,3,4]. Amyloid-beta (Aβ) is a protein that is implicated in AD, one of the most common forms of dementia. Aβ deposition, which can be observed via amyloid positron emission tomography (PET) imaging, is one of the earliest detectable pathological changes and a pathological hallmark of AD. It precedes other biomarkers, such as tau pathology, neuronal injury or neurodegeneration, and cognitive symptoms [5]. Once amyloid plaques start to build up, there is a cascade of events, including the accumulation of tau tangles inside neurons and eventual cell death, leading to brain atrophy, which can be observed through magnetic resonance imaging (MRI). Detecting the presence or predicting the onset of Aβ positivity can, therefore, be instrumental in the early diagnosis and prevention of this debilitating disease.

Numerous studies have employed machine learning and deep learning methodologies for predicting amyloid pathology and other AD phenotypes [1,6,7,8,9,10,11,12,13,14,15,16,17,18,19,20], typically classifying individuals into the categories of cognitively normal (CN), mild cognitive impairment (MCI), and Alzheimer’s disease (AD) [11,12,13,14]. Furthermore, several studies have proposed machine learning frameworks designed to predict the conversion from MCI to AD [15,16,17,18,19], highlighting the potential of these methodologies in forecasting AD progression. Despite these advances, a crucial area of focus remains the early detection of AD pathology, especially in cognitively unimpaired individuals. Aβ deposition, distinguishable before any cognitive impairment becomes apparent, serves as a pivotal biomarker for identifying individuals predisposed to AD. It is noteworthy that cognitively unimpaired individuals may not yet demonstrate significant Aβ deposition and are often categorized as Aβ negatives. However, AD typically harbors an extended preclinical phase where, despite an absence of overt cognitive symptoms, individuals may already possess considerable Aβ deposition [21,22]. Thus, predicting Aβ positivity could significantly expedite AD diagnosis, optimize participant selection for clinical trials of disease-modifying therapies, and facilitate proactive monitoring and potential early intervention. Many researchers are increasingly targeting the early stages and the preclinical phase of AD in an attempt to curtail disease progression [1,8,20,23].

Various biomarkers, including demographic traits, genetic factors, and MRI imaging features, are key tools in predicting Aβ positivity [24,25]. Age, a significant demographic factor, is intrinsically linked with the risk of developing AD. As age advances, the probability of both AD and other dementia forms, as well as Aβ plaque accumulation, increases. Sex is another demographic characteristic of note, with women statistically more likely to develop AD compared to men, a fact which is an active area of investigation. Educational attainment, gauged as years of formal education completed, is associated with AD risk. Those with higher education levels often exhibit a lower risk of AD, potentially attributed to a bolstered cognitive reserve, which allows for increased tolerance of brain damage before dementia symptoms manifest.

Genetic factors, particularly the apolipoprotein E (APOE) ϵ4 allele, have a significant role in AD risk prediction [26,27,28]. Carriers of this allele are at elevated risk of developing AD and often experience earlier onset of symptoms. This allele is believed to influence AD by modifying Aβ processing or clearance in the brain. MRI features, including brain atrophy and other structural changes linked to AD, offer powerful predictive tools for Aβ positivity [29,30]. Changes such as specific regional brain shrinkage, ventricular expansion, and alterations in white matter integrity can be detected by MRI. The combined utilization of these biomarkers offers a comprehensive approach to predicting Aβ positivity. The multi-faceted strategy facilitates earlier, more precise diagnosis; improved prognostic predictions; and the potential for personalized treatment plans. It can also guide the design of clinical trials and the development of new therapeutic interventions, underlining the enhanced predictive model offered by their combined use.

Effective capture of the collective power of these biomarkers for the efficient diagnosis and prognosis of AD can be achieved through graph-based machine learning, particularly graph neural networks (GNNs). GNNs have made significant strides in the healthcare sector, modeling interactions between biological entities, predicting potential disease-associated genes, constructing patient similarity networks, and even playing a crucial role in drug discovery [31,32,33,34,35,36,37,38,39,40,41]. GNNs have been utilized to model intricate correlations between multiple biomarkers, such as genetic, clinical, and neuroimaging features, offering valuable insights into the underlying mechanisms of AD progression [42,43,44,45,46]. The strength of GNNs lies in their ability to interpret the interconnections between brain regions and the impacts of changes in these regions on AD progression. These capabilities can enhance understanding of the complex disease trajectory, allowing for more precise prediction of AD prognosis.

One such tool that has proven to be powerful in graph-based deep learning is the subset of GNNs known as graph convolutional networks (GCNs) [47]. They extend the concept of convolutional operations from regular, grid-like structures—typical in images—to irregular graph structures. Notably, GCNs have been shown to be useful in disease prediction, particularly for autism spectrum disorder and AD [38]. They operate on a population graph, where nodes represent individuals and edges symbolize similarity in certain characteristics, thereby facilitating the deciphering of population-level patterns and individual variations in brain images.

Despite these advances, their inherent black-box nature poses a challenge due to limited transparency. This opacity can hinder understanding of the models’ internal decision-making processes—a significant concern in the medical field, where model interpretability is crucial. To combat this issue, researchers are turning to explainable artificial intelligence (XAI) to foster more comprehensible and transparent GNNs. Various techniques, such as GradCAM-based explanation [48,49], PGExplainer [50], PGMExplainer [51], XGNN [52], and GNNExplainer [53], have been developed to enhance the interpretability of GNNs. However, the use of explainable GNNs remains largely restricted to medical image analysis [54,55,56] and drug discovery [57,58,59,60], suggesting a need for broader application and integration. Moreover, the comprehensive prioritization of personalized biomarkers, crucial for personalized medicine in AD diagnosis and treatment, is a largely unexplored area.

**Figure 1:**
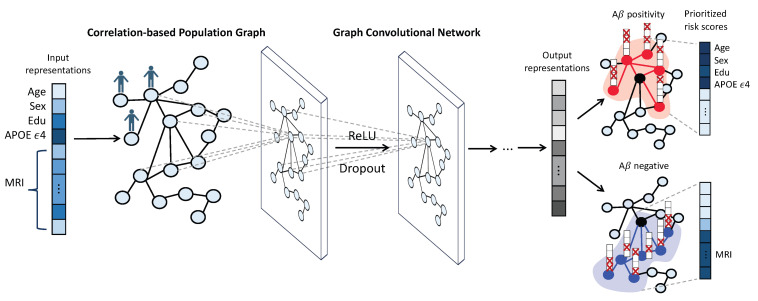
Overview of the proposed model. First, we construct a population graph in which the vertices represent individuals and are characterized by demographic features (age, sex, years of education), genetic information (APOE ϵ4 status), and MRI imaging features (average cortical thickness values for 69 brain regions of interest (ROIs). Edges are assigned when there is a high correlation between a pair of individuals. Next, we employ graph convolutional networks (GCNs) to analyze the population graph and predict the Aβ positivity for each individual. Finally, GNNExplainer provides an explanation for each prediction, optimizing a subgraph of the individual’s neighborhood and identifying a set of crucial features for the prediction. For each individual, we prioritize the top 10 personalized biomarkers by assigning risk scores based on the average importance values obtained from their neighbors.

This study aims to bridge this gap by leveraging GCNs [47] to offer accurate predictions alongside interpretable results, thus contributing to a more holistic understanding of individual AD prognoses. This study is motivated by the hypothesis that cohorts with analogous clinical or neuroimaging characteristics may show a correlation that extends beyond the influence of prevalent biomarkers, such as the APOE genotype. Thus, we can build a population graph where nodes symbolize individuals at risk and edges depict similarities in demographic, genetic, and neuroimaging attributes. Our study highlights the utility of GCNs in predicting Aβ positivity, a crucial early indicator of AD, by demonstrating our proposed correlation-based population graph of cognitively unimpaired individuals. Furthermore, we utilize GNNExplainer [53], an explainable GNN model, which optimizes a subgraph within an individual’s neighborhood and pinpoints a set of crucial features integral for the prediction. For each individual, we prioritize personalized AD risk factors, allocating risk scores derived from the average importance values garnered from their neighbors. This elucidation process further unveils a significant variation in the biomarkers identified for AD prognosis across different sample groups. The overview of the proposed model is illustrated in Figure 1. The main contributions of this study include:

- We demonstrate the effectiveness of using graph neural networks on population graphs for early AD diagnosis in cognitively unimpaired individuals;
- We provide explanations of graph neural network predictions, offering sample-level interpretations using demographic and neuroimaging features;
- We prioritized personalized risk factors for AD by explaining graph neural network predictions, thereby characterizing groups of individuals based on their risk factors in AD prognosis.

## 2. Materials and Methods

### 2.1. Dataset

In this study, we leveraged the Alzheimer’s Disease Neuroimaging Initiative (ADNI) GO/2 dataset (adni.loni.usc.edu, accessed on 21 May 2023). The ADNI was launched in 2003 as a public–private partnership led by principal investigator Michael W. Weiner, MD. The primary goal of ADNI is to test whether serial MRI, PET, other biological markers, and clinical and neuropsychological assessment can be combined to measure the progression of mild cognitive impairment (MCI) and early Alzheimer’s disease (AD). For up-to-date information, see www.adni-info.org (accessed on 21 May 2023). Our selection targeted individuals identified as either cognitively normal (CN) or with mild cognitive impairment (MCI) status. We utilized a total of 506 samples from the ADNI cohort encompassing 214 CN and 292 MCI samples, with each sample characterized by 73 features.

These features included 3 demographic aspects (age, sex, and years of education), APOE ϵ4 status, and 69 neuroimaging features derived from MRI scans. The APOE ϵ4 status, denoted by the count of ϵ4 alleles (0, 1, or 2), functions as a critical genetic biomarker associated with elevated AD risk, with two ϵ4 alleles conferring the highest susceptibility. The neuroimaging features derived from MRI provide a thorough representation of neuroanatomical alterations, encompassing metrics such as cortical thickness. In this study, we utilized quantitative ADNI MRI data. The quantitative MRI data specifically represent the cortical thickness from T1-weighted MRI images obtained from the University of California, San Francisco, and archived at the LONI Image and Data Archive (IDA). We used average cortical thickness values for each of the 69 brain regions of interest (ROIs). A comprehensive description of the MRI image data acquisition process can be found here: https://adni.loni.usc.edu/methods/mri-tool/mri-analysis/ (accessed on 21 May 2023). As a result, there were 69 distinct numerical MRI features available for each individual.

Our study primarily focuses on predicting Aβ positivity in cognitively unimpaired individuals. The levels of Aβ were quantified from ${}^{18}$F-florbetapir PET scans, which specifically bind to Aβ plaques present in the brain. The measurements of Aβ levels were obtained from the LONI Image and Data Archive (IDA) at the University of California, Berkeley. The burden of Aβ deposits was evaluated using the averaged value of the standardized uptake value ratio (SUVR). A detailed description of the PET image analysis method can be found here: https://adni.loni.usc.edu/methods/pet-analysis-method/ (accessed on 21 May 2023). If the averaged value exceeded a cutoff of 1.11, the individual was classified as Aβ-positive, indicative of AD pathology [61,62]. The ADNI cohort contained 291 Aβ-negatives and 215 Aβ-positives. Summary statistics of the clinical features for the CN and MCI samples are presented in Table 1.

### 2.2. Population Graph Construction

In our study, we constructed a graph denoted by G=(V,E), where the vertex set $V=(v_{1},\ldots ,v_{n})$ signifies the collection of individuals. Each vertex $v_{i}$ is associated with the *i*-th individual and is characterized by a feature vector $x_{v_{i}}$, which is composed of demographic and neuroimaging features. Specifically, for each individual, we concatenate features such as age, sex, years of education, APOE ϵ4 status (0, 1, or 2), and quantitative MRI imaging features (average cortical thickness values for 69 brain ROIs), resulting in a 73-feature vector. The set E denotes the collection of undirected edges linking the vertices in V.

Pearson’s correlation coefficient and associated *p*-values are computed for each vertex pair’s feature vectors $x_{v}$ and $x_{w}$. These metrics elucidate the magnitude, direction, and statistical significance of the linear relationship between the data linked to each vertex pair. Following this calculation, we rank all vertex pairs using two criteria: the absolute correlation coefficient value (in descending order) and the *p*-value (in ascending order). We identify the top *M* pairs, those exhibiting the highest absolute correlation and lowest *p*-values, and assign them edges, $e_{vw}=\left(v,w\right)\in E$. These assigned edges signify the most statistically significant and the strongest correlations within our population graph.

We introduced the proposed model, which employs GCNs and was termed GCN-corr, to this correlation-based population graph. Its performance was contrasted with an equivalently sized graph but with edges assigned randomly termed GCN-random. This comparison elucidated the advantages of utilizing a correlation-based graph for predicting Aβ positivity in cognitively unimpaired individuals as opposed to a graph with randomly assigned edges. In the GCN-corr model, edges mirror the correlations between individual nodes, thus enabling more precise prediction congruent with the population structure.

Through an ablation study, we ascertained the optimal number of edges (*M*) by manipulating the network density. This density, calculated as $\frac{2M}{N(N-1)}$, represents the proportion of actual edges compared to the maximum possible in a fully connected network of N nodes. We varied the density from a sparse 1% connectivity network to a maximally interconnected network (100% connectivity), with each increment representing a 10% increase. This methodology aids in striking an equilibrium between network complexity and predictive accuracy, thereby refining the graph’s structure for enhanced prediction efficacy using the GCN model. Additional discussions on the ablation study are expounded upon in the Supplementary Materials.

### 2.3. Graph Convolutional Networks

In this study, we leveraged graph convolutional networks (GCNs), which were proposed by [47], with a population graph G to discern the connections between the demographic and neuroimaging features of individuals. This study addresses the problem of predicting Aβ positivity as a node (individual)-level prediction task within the GCN model. An adjacency matrix $A\in {\left[0,1\right]}^{N\times N}$ representing pairwise correlations between nodes in a population graph G, a feature matrix $X\in R^{N\times p}$, and labels $y\in {\left[0,1\right]}^{N}$ (Aβ positivity) are used as input to train the model.

GCNs are designed to learn robust node representations by aggregating information from the local neighborhoods within the graph [47]. The core operation in GCNs, graph convolution, operates as a message-passing mechanism that facilitates information exchange between adjacent nodes. Within each GCN layer, nodes gather, process messages from their neighbors, and subsequently transform this information using a learnable weight matrix. Diverging from the convolution concept used in convolutional neural networks (CNNs), which apply a filter to localized input data segments, GCNs redefine convolution as the process of aggregation of neighboring node information. This notion retains the integral principle of incorporating local information, a concept fundamental to CNNs, thus justifying its appellation as graph convolution. Within a single GCN layer, the operation can be denoted as:

(1) $$H^{\left(l+1\right)}=\sigma \left({\tilde{D}}^{-\frac{1}{2}}\tilde{A}{\tilde{D}}^{-\frac{1}{2}}H^{\left(l\right)}W^{\left(l\right)}\right)$$

In this equation, $H^{\left(l\right)}$ and $H^{\left(l+1\right)}$ represent the feature matrices at the *l*-th and (l+1)-th layers, respectively, each encapsulating node representations. The matrix $\tilde{A}$, derived by adding the identity matrix *I* (representing self-loops, which are edges from nodes to themselves) to the original adjacency matrix *A*, is referred to as a self-loop inclusive adjacency matrix. The degree matrix of $\tilde{A}$, $\tilde{D}$, is a diagonal matrix representing the degree (number of connections) of each node. The learnable weight matrix at the *l*-th layer, $W^{\left(l\right)}$, serves to transform the aggregated neighbor node information. σ signifies the activation function; in this study, a rectified linear unit (ReLU).

The model is then trained to minimize the loss L using the ADAM optimizer and learn the optimal parameters for the prediction task. The loss L represents a cross-entropy function, which is computed as follows:

(2) $$L=-(w_{y_{0}}y\log\left(h\right)+w_{y_{1}}\left(1-y\right)\log\left(1-h\right))$$

In this equation, *h* denotes the model’s prediction output following softmax activation, and y represents the ground-truth label. Given the higher number of Aβ-negative samples in the cognitively unimpaired individuals dataset, the model could be impacted by class imbalance. To address this issue, we assigned rescaling weights during model training, which are inversely proportional to class frequencies as follows:

(3) $$w_{y_{c}}=\frac{N}{2N_{y_{c}}}$$

Here, *N* represents the total number of samples, while $N_{y_{c}}$ denotes the number of samples belonging to class *c*.

### 2.4. Interpretation with GNNExplainer

GNNExplainer [53] is a model-agnostic method specially designed to elucidate the predictions made by graph neural networks (GNNs), including the GCN model. The primary goal of GNNExplainer is to offer insights into the model’s decision-making process, highlight significant features and relationships within the graph, and foster trust in the model’s predictions.

The GNNExplainer method works by learning to extract a concise subgraph from the original input graph. This subgraph is optimized to best explain the GNN model’s prediction for a specific target node or graph. This is accomplished by formulating an optimization problem where the objective is to minimize the difference between the original GNN model’s prediction and the prediction made using the extracted subgraph. This optimization process involves the use of a binary mask for nodes, edges, and features. This mask determines whether to include or exclude graph elements, contingent on their contribution to the prediction.

In this research, we employed GNNExplainer to provide interpretable explanations for the predictions generated by our GCN model. This tool allowed us to explore individual biomarkers that significantly contribute to the prediction of Aβ positivity. Additionally, we identified distinct groups of individuals who share common biomarkers yet contain unique prioritized features that differentiate them from other groups. We also detailed the characteristics of each group based on their significant biomarkers.

### 2.5. Performance Evaluation

In this study, we formulated a supervised node classification problem, where our objective was to predict the Aβ positivity for each individual within the test sets. We assessed the classification performance by employing stratified five-fold cross-validation repeated 10 times. This method ensured that the ratio of Aβ-positive to Aβ-negative samples was maintained across all sets. During each iteration, we set aside one fold as the test set, while the remaining four folds were randomly divided into an 80% training set and a 20% validation set. We carried out this stratified partitioning to optimize and validate our model. Consequently, the data were split into a 64% training set, a 16% validation set, and a 20% test set. We report the final classification performance as the mean area under the curve (AUC) values over a total of 50 iterations.

## 3. Results

### 3.1. Experimental Setting

In the experiments, we assessed the performance of our proposed correlation-based population graph model (GCN-corr) and compared it with an equivalently sized graph but with edges assigned randomly (GCN-random). Furthermore, to provide a broader perspective on the efficacy of GCN models applied to population graphs, our comparison was not limited to GCN-random and GCN-corr. We expanded our analysis to include a comparison with traditional machine learning algorithms. These encompassed the support vector machine (SVM) using a radial basis function (RBF) kernel, the random forest (RF) classifier, logistic regression (LR) with the L2 penalty (also known as ridge regularization), and the multi-layer perceptron (MLP).

To achieve optimal performance in our analyses, we set certain hyperparameters empirically for both the GCN and the MLP models. The GCN model was structured as a two-layer network, which included a hidden layer comprising 32 units. The model underwent training for a maximum of 550 epochs, with a learning rate set at 0.005. The L2 loss was set to $5\times 10^{-10}$, and a dropout rate of 0.5 was employed to prevent overfitting and enhance generalization. Simultaneously, the MLP model was set up with the same hyperparameters as the GCN model for a fair comparison.

Due to the predominance of Aβ-negative samples in our dataset, we utilized class weights as outlined in Equation (3) to balance the classes. For fairness in comparison, the same rescaling weights were applied to the conventional machine learning models previously mentioned. This adjustment in weights ensured a balanced evaluation of each model’s performance, despite the unequal sample sizes between the Aβ-positive and Aβ-negative groups.

It is worth noting that, although we employed stratified cross-validation, a technique which guarantees a proportionate class distribution across all validation folds, it did not directly influence the model’s learning process. Conversely, class weights were incorporated during model training, conferring more emphasis to the less represented class and thereby ameliorating the effects of imbalanced data. Therefore, these two methodologies, while serving distinct roles, complement each other: stratified cross-validation augments the accuracy of performance estimation, while class weighting refines the model’s capacity to learn from imbalanced datasets.

### 3.2. Performance of Prediction of Aβ Positivity

**Figure 2:**
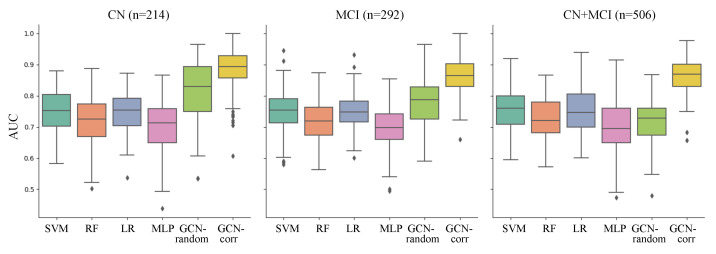
Comparison of the performance of each model (represented in x-axis and different colors) in predicting Aβ positivity across three sample groups: cognitively normal (CN) individuals, those with mild cognitive impairment (MCI), and a combined group (CN + MCI). The performance was measured with the area under the ROC curve (AUC) derived from 10 repetitions of five-fold cross-validation. The compared models are support vector machine using a radial basis function kernel (SVM), random forest (RF) classifier, logistic regression with ridge regularization (LR), multi-layer perceptron (MLP), GCN model on a graph with randomly assigned edges (GCN-random), and GCN model on a correlation-based population graph (GCN-corr).

We evaluated the performance in predicting Aβ positivity with comparative analyses conducted across three distinct groups: cognitively normal (CN) individuals, individuals with mild cognitive impairment (MCI), and a combined group of both CN and MCI individuals. The performances for each model were evaluated across these sample groups and are comprehensively displayed in Table 2 and Figure 2.

Upon analysis, we observed that the GCN models, when applied to the population graph constructed in this study, consistently outperformed the conventional machine learning models across all sample groups. Moreover, the correlation-based population graph model (GCN-corr) consistently demonstrated superior performance compared to the GCN model with randomly assigned edges (GCN-random). The GCN-corr model outperformed all other models across all groups, achieving the highest mean AUCs of 0.8851, 0.8741, and 0.8632 for the CN, MCI, and CN + MCI groups, respectively. This was particularly evident in the combined CN + MCI group, where the performance of the GCN-random model showed a significant drop (0.7160 ± 0.0135), not just in comparison to the GCN-corr model but also against most of the conventional machine learning models. This suggests the potential of GCN models in significantly enhancing the predictive accuracy for Aβ positivity when used with well-crafted population graphs, affirming their proficiency in handling complex biological data.

**Figure 3:**
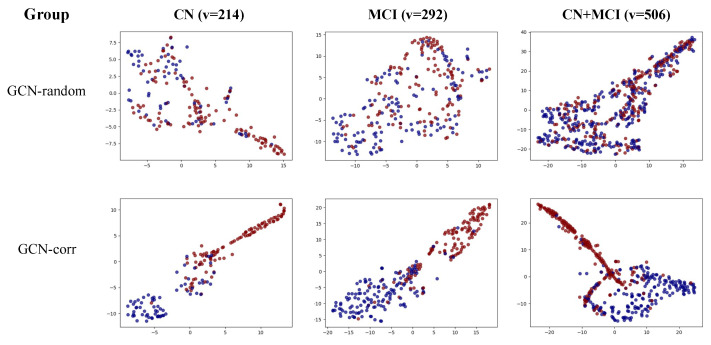
Visual representation of the final node embedding of the GCN-random and GCN-corr models across three groups: cognitively normal (CN), mild cognitive impairment (MCI), and a combined CN + MCI group. Nodes representing Aβ-positive samples are colored blue, while those representing Aβ-negative samples are in red. v represents the number of vertices (individuals).

To further illustrate the proficiency of the GCN model in accurately classifying Aβ-positive and -negative samples within a population graph, we offer a visualization of the final embedding of each GCN model in Figure 3. Notably, the GCN model with randomly assigned edges (GCN-random) demonstrated difficulty differentiating between the two classes across all three groups: cognitively normal (CN), mild cognitive impairment (MCI), and the combined CN + MCI group.

In contrast, the correlation-based population graph model (GCN-corr) effectively distinguished between the two classes across all sample groups, emphasizing the significant contribution of our proposed correlation-based population graph to the enhancement of Aβ positivity prediction using the GCN model.

The strength of these findings lends considerable support to our research hypothesis. It suggests the existence of shared biomarkers within groups of individuals whose demographic and neuroimaging features strongly correlate, thus positively influencing Aβ positivity prediction and enriching our understanding of AD prognosis. These results also suggest variability in AD risk factors across different individual groups, hinting at the potential benefits of tailoring prediction models and preventative strategies.

### 3.3. Interpreting Predictions of Graph Neural Networks

In our endeavor to understand the intricate interplay between demographic and neuroimaging features, we utilized GNNExplainer. This tool helped us identify key biomarkers that significantly contribute to the prediction of Aβ positivity within GCN models. This investigation deepens our understanding of the model’s decision-making process and bolsters our confidence in its predictive capabilities.

**Figure 4:**
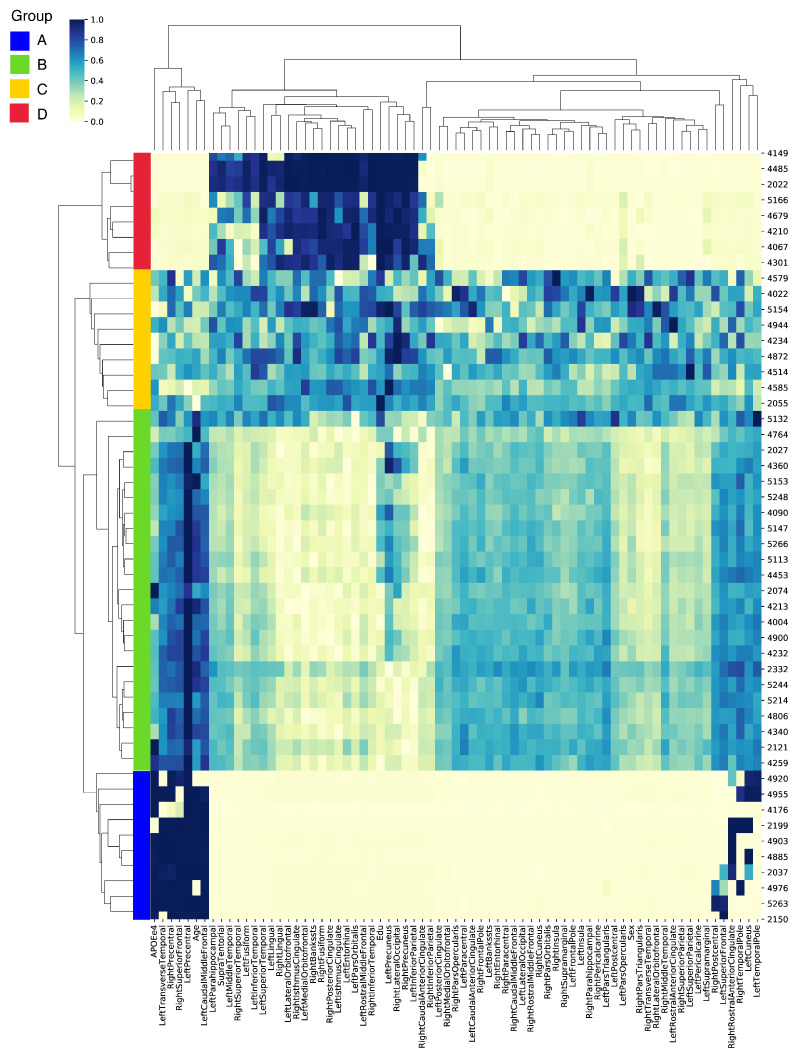
Heatmap visualization of the prioritized biomarkers derived from the GCN model with a correlation-based population graph. Each row represents an individual, and each column represents a biomarker. The color intensity indicates the importance score of each biomarker for Aβ-positivity prediction. The individuals and biomarkers are clustered feature-wise and sample-wise, revealing four distinct groups (A, B, C, and D) based on their important biomarkers.

Figure 4 offers a visual representation of these prioritized biomarkers and their associated importance scores for each individual. For an improved visualization, we randomly selected 50 individuals from the test set that delivered the most accurate predictions across all cross-validation splits. Notably, these samples were correctly classified, which facilitated the identification of the most influential biomarkers for accurate predictions. To further enhance data interpretability, we employed both feature-wise and sample-wise clustering. This analysis led to the identification of four distinct groups (A, B, C, and D), each distinguished by the significance of their biomarkers. We delineated the top 10 biomarkers based on their averaged importance scores, uncovering unique patterns of significance across the four groups, as detailed in Table S1.

In group A, the left precentral gyrus, known for its involvement in motor function, emerged as the most significant biomarker with an average score of 0.9954. Additional notable biomarkers included the right precentral gyrus, the APOEe4 gene variant—associated with an increased risk of AD—and the left caudal middle frontal gyrus, all scoring above 0.89. In group B, the left precentral gyrus was also the top biomarker. However, the demographic feature of age was highlighted, receiving an average score of 0.7768. This underlines the well-established link between advancing age and increased risk of AD. In group C, the left precuneus, a brain region involved in episodic memory, stood out as the most significant biomarker with a score of 0.7137. This group also prioritized demographic features, such as the level of education (years) and the individual’s sex, with scores of 0.6725 and 0.6160, respectively. These results may indicate a potential influence of educational attainment and biological sex on the disease’s onset and progression. Group D highlighted the value of education (years) as the top biomarker, scoring a near-perfect 0.9970. This aligns with the cognitive reserve theory, which suggests that higher levels of education may offer a protective effect against cognitive decline. Neuroimaging biomarkers, such as the right precuneus and left pars orbitalis, also held high priority in this group.

Taken together, these findings underscore the complex interaction between structural brain changes and demographic factors in predicting Aβ positivity. They shed light on the heterogeneity of the disease, revealing different progression patterns across unique groups.

## 4. Discussion

Our study illuminates the potential value of applying GCNs within a correlation-based population graph for enhanced AD prognosis. The superior performance of this model underscores the efficacy of harnessing the inherent structure and correlations in the data to augment predictive accuracy.

Our findings comprehensively demonstrate that a population graph built on correlations rather than random assignment can significantly elevate the model’s ability to discern patterns and predict accurately. This suggests that the interplay between demographic and neuroimaging features is not random; instead, it exhibits a specific correlation structure that is instrumental for the prediction task.

Moreover, the improved performance lends credence to the hypothesis of the existence of common biomarkers among groups of individuals who exhibit high correlations in their demographic and neuroimaging features. This implies that different groups may possess distinctive sets of biomarkers that are particularly indicative of Aβ positivity, thereby illuminating the heterogeneity of AD progression.

Thus, our research underscores the potential of utilizing correlation-based population graphs in tandem with GCN models for a more nuanced and effective AD prognosis. This approach could potentially inform the development of more personalized, precise therapeutic strategies.

Future research could build upon our findings and explore several promising directions. This could include enriching the GCN model by incorporating diverse data sources, such as genetic, proteomic, and lifestyle factors, alongside demographic and neuroimaging features, to enhance the predictive performance. Transitioning from a cross-sectional model to one that exploits longitudinal data could offer a more dynamic understanding of AD progression. A deeper exploration of model interpretability and biomarker validation could provide greater transparency for the decision-making process, ensuring the identified biomarkers’ relevance. Understanding the unique contributions of biomarkers to AD progression in different groups could inform the development of personalized treatment strategies. Lastly, the proposed framework in this study could be expanded to other neurological disorders or complex diseases, potentially offering valuable insights into disease mechanisms and therapeutic targets.

## Figures and Tables

**Table 1:** Demographic and neuroimaging characteristics of cognitively normal (CN) and mild cognitive impairment (MCI) groups from the Alzheimer’s Disease Neuroimaging Initiative (ADNI) dataset.

	CN (N = 214)	MCI (N = 292)
Age	74.18 ± 6.14	73.14 ± 7.48
Sex	96 F/118 M	173 F/119 M
Education (years)	16.7 ± 2.54	16.13 ± 2.64
APOE ϵ4	144 neg/70 pos	173 neg/119 pos
Aβ positivity	139 neg/75 pos	152 neg/140 pos

N: Number of samples; F: Female; M: Male; pos: Positive; neg: Negative

**Table 2:** Summary of the performance of each model in predicting Aβ positivity across three sample groups: cognitively normal (CN) individuals, those with mild cognitive impairment (MCI), and a combined group (CN + MCI). The performance metrics were computed as the mean area under the ROC curve (AUC), along with a 95% confidence interval, derived from 10 repetitions of five-fold cross-validation. The highest performing result for each sample group is highlighted in bold text.

	AUC (Mean ± 95% CI)		
Model	CN	MCI	CN + MCI
SVM (RBF)	0.7515 ± 0.0131	0.7531 ± 0.0137	0.7537 ± 0.0129
RF	0.7205 ± 0.0143	0.7226 ± 0.0140	0.7238 ± 0.0134
LR (ridge)	0.7490 ± 0.0129	0.7480 ± 0.0112	0.7500 ± 0.0144
MLP	0.7009 ± 0.0158	0.7013 ± 0.0137	0.7009 ± 0.0159
GCN-random	0.8110 ± 0.0185	0.7768 ± 0.0153	0.7160 ± 0.0135
GCN-corr	**0.8851 ± 0.0154**	**0.8741 ± 0.0114**	**0.8632 ± 0.0115**

## Data Availability

Data used in the preparation of this article were obtained from the Alzheimer’s Disease Neuroimaging Initiative (ADNI) database (adni.loni.usc.edu, accessed on 21 May 2023). As such, the investigators within the ADNI contributed to the design and implementation of the ADNI and/or provided data but did not participate in analysis or writing of this report. A complete listing of ADNI investigators can be found at: http://adni.loni.usc.edu/wp-content/uploads/how_to_apply/ADNI_Acknowledgement_List.pdf (accessed on 21 May 2023).

## Supplementary Materials

The following supporting information can be downloaded at: https://www.mdpi.com/article/10.3390/bioengineering10060701/s1, Figure S1: Performances of the GCN-corr and GCN-random models as network density varies from a sparse 1% connectivity to a fully connected 100% connectivity in increments of 10%. Performance metrics were calculated as the mean area under the ROC curve (AUC), with a 95% confidence interval, derived from 10 repetitions of five-fold cross-validation. The confidence interval’s upper and lower bounds are visually represented as a shaded area surrounding the line; Figure S2: Visual representation of the final node embedding of the GCN-random and GCN-corr models at selected network densities of 1%, 10%, and 50%. These densities were chosen to provide a clear comparison across varying levels of network densities. Nodes representing Aβ-positive samples are colored blue, while those representing Aβ-negative samples are in red; Table S1: The top 10 prioritized biomarkers from demographic and neuroimaging features, along with their corresponding averaged feature importance scores, listed in descending order. The biomarkers are divided into four groups (A–D) based on the results of heatmap clustering.
