# Supplementary material for: Personalized Explanations for Early Diagnosis of Alzheimer’s Disease Using Explainable Graph Neural Networks with Population Graphs
Source: Bioengineering (Basel). 2023 Jun 8;10(6):701. doi: 10.3390/bioengineering10060701 (PMC10295378; doi:10.3390/bioengineering10060701)
Supplement: Supplementary file 1 [file bioengineering-10-00701-s001.zip › Supplementary Materials.pdf]

Supplementary Materials: Personalized Explanations for Early Diagnosis of Alzheimer's Disease Using Explainable Graph Neural Networks with Population Graphs

So Yeon Kim [1,2]

[1] Department of Artificial Intelligence, Ajou University, Suwon 16499, Republic of Korea; jebi1771@ajou.ac.kr
[2] Department of Software and Computer Engineering, Ajou University, Suwon 16499, Republic of Korea

1. Ablation Study

1.1. Optimizing Network Density of Graph Neural Networks

To determine the optimal number of edges (M) of a population graph, we conducted an ablation study to assess performance outcomes associated with varying network densities, as illustrated in Figure S1. Moreover, we visualized the final embeddings generated by each GCN model in Figure S2. Our analysis demonstrated that GCN models with randomly assigned edges significantly underperformed compared to the GCN-corr, irrespective of network density. This is further evidenced by Figure S2, highlighting the inability of such models to effectively differentiate between Aβ positive and negative samples. Conversely, the GCN-corr model demonstrated superior performance and more informative embeddings. These results affirm the effectiveness of our proposed method for population graph construction. Notably, the GCN-corr model achieved its best performance at a network density of merely 1%, but it was also able to maintain the correlation structure to some extent at higher densities. These findings highlights the crucial role of careful network density selection of population graphs in designing efficient GCN models.

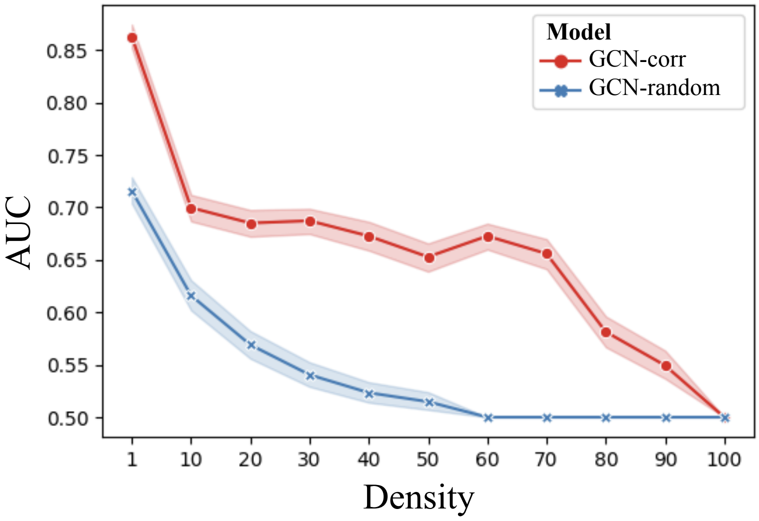

Figure S1. Performances of the GCN-corr and GCN-random models, as network density varies from a sparse 1% connectivity to a fully connected 100% connectivity network, in increments of 10%. Performance metrics are calculated as the mean Area Under the ROC Curve (AUC) with a 95% confidence interval, derived from 10 repetitions of 5-fold cross-validation. The confidence interval's upper and lower bounds are visually represented as a shaded area surrounding the line.

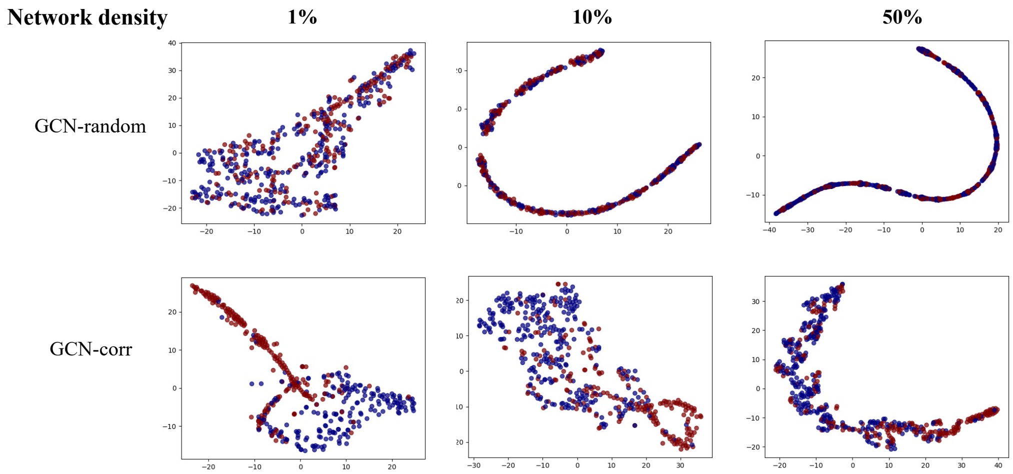

Figure S2. Visual representation of the final node embedding of the GCN-random and GCN-corr models at selected network densities of 1%, 10%, and 50%. These densities were chosen to provide a clear comparison across varying levels of network densities. Nodes representing Aβ positive samples are colored blue, while those representing Aβ negative samples are in red.

2. Supplementary Table

Table S1. The top-10 prioritized biomarkers from demographic and neuroimaging features, along with their corresponding averaged feature importance scores, listed in descending order. The biomarkers are divided into four groups (A-D) based on the results of heatmap clustering.

Group	Feature	Averaged Score
A	Left Precentral	0.9954
	Right Precentral	0.9027
	APOE $\epsilon 4$	0.8985
	Left Caudal Middle Frontal	0.8951
	Right Superior Frontal	0.8116
	Age	0.7965
	Left Transverse Temporal	0.6984
	Left Cuneus	0.4502
	Right Rostral Anterior Cingulate	0.4399
	Right Temporal Pole	0.3009
B	Left Precentral	0.9516
	Age	0.7768
	Left Caudal Middle Frontal	0.7382
	Right Superior Frontal	0.7109
	Right Precentral	0.7048
	Right Temporal Pole	0.6513
	Left Transverse Temporal	0.6502
	Left Temporal Pole	0.6380
	Left Superior Frontal	0.6227
	Left Cuneus	0.5842
C	Left Precuneus	0.7137
	Right Isthmus Cingulate	0.6937
	Right Lateral Occipital	0.6729
	Education (years)	0.6725
	Left Superior Parietal	0.6614
	Right Superior Temporal	0.6404
	Right Inferior Temporal	0.6382
	Left Rostral Anterior Cingulate	0.6276
	Left Isthmus Cingulate	0.6268
	Sex	0.6160
D	Education (years)	0.9970
	Right Precuneus	0.9804
	Left Precuneus	0.9762
	Left Pars Orbitalis	0.9749
	Right Posterior Cingulate	0.9672
	Left Entorhinal	0.9601
	Right Fusiform	0.9523
	Left Medial Orbitofrontal	0.9451
	Right Lateral Occipital	0.9343
	Right Isthmus Cingulate	0.9228
