# Supplementary figures and images for: Personalized Explanations for Early Diagnosis of Alzheimer’s Disease Using Explainable Graph Neural Networks with Population Graphs
Source: Bioengineering (Basel). 2023 Jun 8;10(6):701. doi: 10.3390/bioengineering10060701 (PMC10295378; doi:10.3390/bioengineering10060701)

# Network density

1%

10%

50%

GCN-random

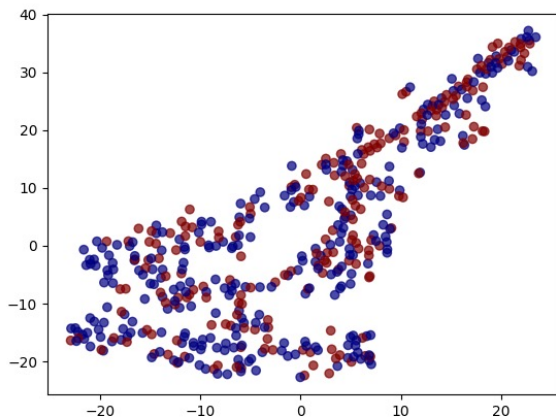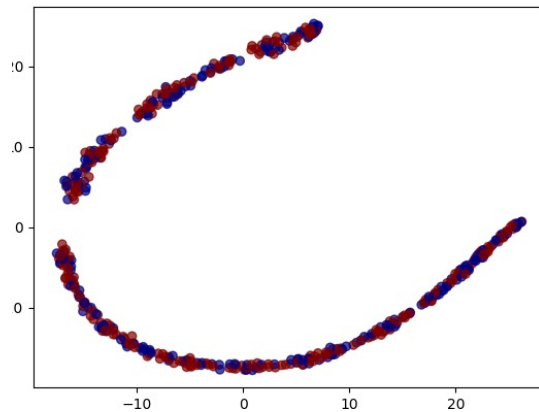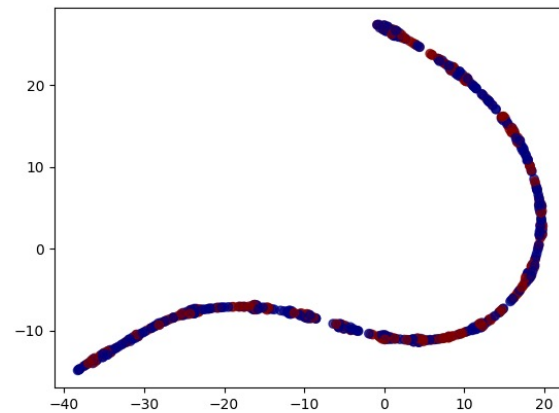

GCN-corr

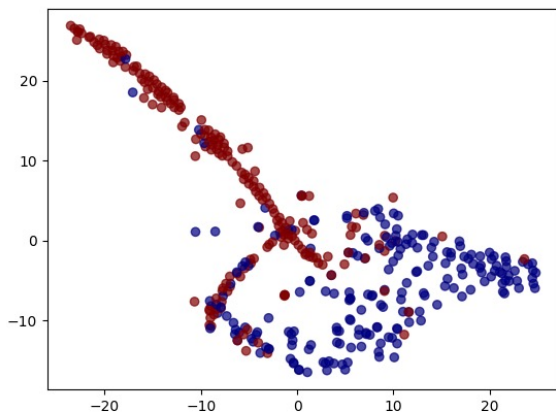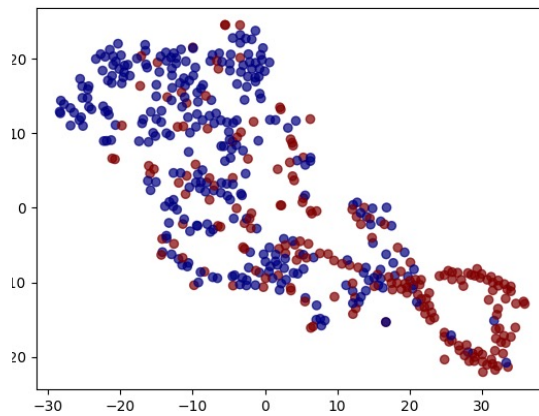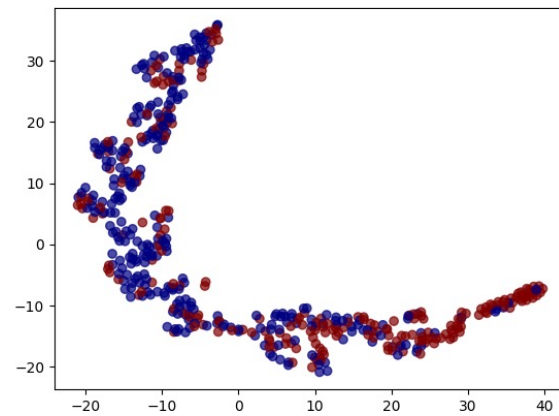

Supplement: Supplementary file 1 [file bioengineering-10-00701-s001.zip › fig_gcn_density.pdf]

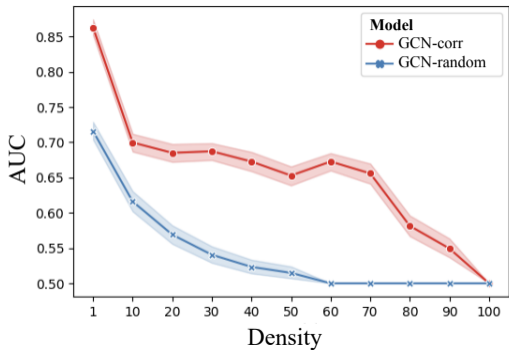

Supplement: Supplementary file 1 [file bioengineering-10-00701-s001.zip › fig_perf_density.pdf]
